# Knowledge, attitudes, and practices of healthcare professionals regarding palliative care family meetings: a multicenter cross-sectional study

**DOI:** 10.3389/fpubh.2026.1902540

**Published:** 2026-08-21

**Authors:** Chunjin Lin, Xiaona Zheng, Fang Zhu, Chengying Li, Yifang Guo, Fan Lin, Pengli Zhu

**Affiliations:** 1 Shengli Clinical Medical College of Fujian Medical University, Fuzhou, Fujian, China; 2 Department of Geriatric Medicine, Fuzhou University Affiliated Provincial Hospital, Fuzhou, Fujian, China; 3 Department of Nursing Science, Faculty of Medicine, University Malaya, Kuala Lumpur, Malaysia; 4 Department of Medical Oncology, Clinical Oncology School of Fujian Medical University, Fujian Cancer Hospital, Fuzhou, Fujian, China; 5 Department of Oncology, The First Affiliated Hospital of Fujian Medical University, Fuzhou, Fujian, China; 6 Department of Oncology, Xiamen Haicang Hospital, Xiamen, Fujian, China; 7 Fujian Provincial Center for Geriatrics, Fuzhou University Affiliated Provincial Hospital, Fuzhou, Fujian, China; 8 Fujian Key Laboratory of Geriatrics, Fuzhou, Fujian, China; 9 Fujian Provincial Institute of Clinical Geriatrics, Fuzhou, Fujian, China

**Keywords:** cross-sectional study, family meeting, health knowledge, attitude, practice, healthcare professionals, palliative care

## Abstract

**Background:**

To assess the knowledge, attitudes, and practices (KAP) of healthcare professionals (HP) regarding palliative care family meetings and to identify factors independently associated with practice level.

**Methods:**

This multicenter cross-sectional study was conducted among 401 healthcare professionals across four institutions in Fujian Province between January and February 2026. Data were collected using a validated self-designed questionnaire encompassing demographic characteristics and KAP scores. Multivariable logistic regression identified factors independently associated with practice level, and structural equation modeling (SEM) examined whether attitude mediated the knowledge-practice relationship.

**Results:**

The mean scores were 14.35 ± 5.95 for perceived knowledge (55.2% of maximum), 42.63 ± 5.92 for attitude (85.3%), and 24.96 ± 8.93 for practice (71.3%). Multivariable analysis identified knowledge score (OR = 1.088, 95%CI: 1.046–1.132, *p* < 0.001), attitude score (OR = 1.063, 95%CI: 1.023–1.104, *p* = 0.002), and monthly income <5,000 RMB versus >10,000 RMB (OR = 0.448, 95%CI: 0.217–0.923, *p* = 0.029) as independent associates of proactive practice. SEM indicated that attitude partially mediated the perceived knowledge-practice relationship (indirect effect *β* = 0.052, *p* = 0.003), with knowledge also showing a significant direct association with practice (*β* = 0.277, *p* = 0.010).

**Conclusion:**

Healthcare professionals in Fujian Province demonstrated limited perceived knowledge but positive attitudes and proactive practices toward palliative care family meetings. Both perceived knowledge and attitude were independently associated with practice, with attitude serving as a partial mediator. Targeted training programs and institutional support are needed to translate positive attitudinal endorsement into consistent, high-quality practice.

## Introduction

Palliative care, as defined by the World Health Organization, aims to improve the quality of life of people with life-threatening illness and their families through the prevention and relief of suffering, including attention to physical, psychosocial, and spiritual needs (1, 2). Within this broader framework, family meetings represent a practical multidisciplinary tool for delivering person- and family-centered care (3, 4). Palliative care family meetings are structured conversations that bring together healthcare professionals, patients, and family members to discuss care goals, treatment priorities, decision-making, and ongoing support needs (3, 4). They are increasingly recognized as a core communication strategy in palliative care because they help align care with patient values, facilitate shared decision-making, clarify goals of care, and support families during serious illness and end-of-life care (5–7). Thus, structured family meetings provide a practical means of integrating communication, shared decision-making, and multidisciplinary support into palliative care delivery.

However, the routine implementation of structured family meetings varies considerably across healthcare systems and resource settings. In high-income countries such as the United States and Australia, family meetings are widely regarded as best practice in palliative, hospice, and intensive care settings and are supported by systematic evidence and structured clinical guidance (2). In China and other comparable middle-income settings, palliative care services are expanding but remain unevenly distributed, and structured family meetings have not yet been routinely integrated into standard care pathways (8–10). Existing evidence suggests that end-of-life communication is often informal and fragmented, with implementation constrained by heavy workloads, limited communication training, insufficient institutional support, and cultural factors such as family-centered decision-making norms and death-related taboos. As a result, family meetings may occur in an *ad hoc* rather than standardized manner, highlighting an important gap in current palliative care practice (9, 11, 12). These barriers collectively produce an environment in which family meetings, when they occur, are largely *ad hoc*, inconsistently structured, and insufficiently supported by training, staffing, or organizational policy. Addressing these gaps is essential for advancing equitable, high-quality palliative care across China and comparable resource-constrained yet progressively developing health systems.

Knowledge, attitude, and practice (KAP) studies provide a useful framework for assessing what healthcare professionals know, how they perceive a topic, and how they behave in practice (13–15). Although family meetings are increasingly recognized as a key communication competency in palliative care, evidence on healthcare professionals’ KAP regarding this topic remains limited, particularly in low- and middle-income settings and among multidisciplinary teams (10, 16, 17). Existing studies are largely qualitative or focused narrowly on physician perspectives, leaving the preparation, attitudes, and practices of multidisciplinary teams (including nurses, social workers, and clinical psychologists) substantially underexplored (7, 18, 19). Moreover, the interrelationship among KAP components, specifically whether attitudes mediate the translation of knowledge into practice, has not been empirically tested. This gap is consequential: without understanding the mechanisms linking knowledge, attitude, and behavior, interventions may target only one component and fail to achieve sustained practice change.

Therefore, this study aimed to assess healthcare professionals’ KAP regarding palliative care family meetings in Fujian Province, China. It also examined whether attitudes mediate the relationship between knowledge and practice within the KAP framework. The findings are intended to inform targeted training programs and institutional strategies to strengthen family meeting implementation in China and comparable settings.

## Materials and methods

### Study design and setting

This multicenter cross-sectional study was conducted between January and February 2026 at four medical institutions in Fujian Province: Fujian Cancer Hospital, Union Hospital Affiliated to Fujian Medical University, The First Affiliated Hospital of Fujian Medical University, and Xiamen Haicang Hospital. The participating hospitals were selected by convenience, based on an existing regional collaboration network and the feasibility of survey implementation. Clinical departments were identified according to previous literature, local diagnostic and care pathways for palliative care, and the research team’s clinical experience, with the aim of including all departments in which healthcare professionals might encounter terminally ill patients and their families or participate in palliative care family meetings (e.g., oncology, geriatrics, intensive care, emergency care, and other relevant specialties).

### Ethical considerations

The study was approved by the Ethics Committee of Fujian Cancer Hospital (approval #K2026-01-002). All participants provided electronic informed consent prior to completing the questionnaire.

### Participants

Eligible participants were licensed healthcare professionals (including registered physicians, registered nurses, social workers, clinical psychologists, and rehabilitation therapists) who could understand the study purpose and questionnaire and provided informed consent. Individuals on long-term, maternity, or suspension leave during the study period were excluded, as were incomplete questionnaires and those failing quality-control checks.

### Sample size

The minimum required sample size was estimated using Cochran’s formula for survey studies (20): n = Z^2^ × p(1 − p) / d^2^, where Z is the confidence coefficient, p is the estimated proportion, and d is the margin of error. Assuming a 95% confidence level (Z = 1.96), a conservative proportion (*p* = 0.5), and a precision of 5%, the estimated minimum sample size was 385 participants.

### Questionnaire development

The questionnaire was developed based on a systematic review of published literature and relevant clinical guidelines (1, 2, 4, 19). A panel of six domain experts, comprising two palliative care physicians, two certified palliative care nurses, one medical social worker, and one clinical psychologist, all with more than 5 years of experience in palliative care or end-of-life communication, independently evaluated an initial draft for item relevance, content coverage, clarity, cultural appropriateness, and clinical feasibility. Based on their recommendations, items were refined for clarity and comprehensiveness, and the instrument structure was standardized across dimensions.

A pilot study was subsequently conducted with 39 participants to assess reliability and validity. The overall Cronbach’s *α* was 0.920 (knowledge: 0.960; attitude: 0.885; practice: 0.917), indicating good internal consistency across subscales. The Kaiser-Meyer-Olkin (KMO) value was 0.939 (*p* < 0.001), and confirmatory factor analysis (CFA) demonstrated acceptable model fit (Supplementary Table S1). All factor loadings were statistically significant (p < 0.001); loadings for knowledge and practice items were consistently high (>0.85), while attitude items A6 and A7 exhibited lower loadings (~0.62), indicating potential areas for future instrument refinement (Supplementary Table S2; Supplementary Figure S1).

### Questionnaire

The finalized questionnaire, administered in Chinese, comprised four components: (1) demographic and professional characteristics; (2) knowledge; (3) attitude; and (4) practice (see Additional File: Questionnaire). The knowledge dimension included 13 items (K1-K13), scored 0 (not familiar), 1 (have heard of it), or 2 (very familiar), with a total range of 0–26. Thus, this scale captured self-reported familiarity with key concepts and procedures related to palliative care family meetings rather than objectively tested knowledge or competence. The attitude dimension comprised 10 items assessed on a five-point Likert scale from 1 (strongly disagree) to 5 (strongly agree), with a total range of 10–50. The practice dimension included 7 items scored from 1 (never) to 5 (always), with a total range of 7–35. An additional item (P8) assessed perceived barriers to implementing family meetings and was analyzed separately. Following established conventions in KAP research, scores below 70% of the maximum possible score were classified as insufficient, while scores of 70% or above were classified as good (13, 21). The 70% cut-off is widely used in KAP studies as a pragmatic criterion to distinguish between suboptimal and relatively satisfactory levels of knowledge, attitudes, and practices, corresponding approximately to a “passing” level of performance rather than mastery.

### Sampling and recruitment

Uniformly trained research assistants or departmental liaisons at each site coordinated participant recruitment under the guidance of the research team. Within each participating hospital, departmental liaisons and research assistants, working under the guidance of the research team, helped identify eligible healthcare professionals based on professional qualifications, current working status, and department affiliation. Recruitment used an open, voluntary convenience sampling approach among accessible, eligible staff. The questionnaire was administered electronically via a QR code that was disseminated through multiple channels, including departmental WeChat groups, onsite departmental meetings, and provincial palliative care training events.

Departmental liaisons facilitated the distribution of the survey link but did not designate specific individuals to participate, did not require participation, and did not have access to individual responses. All potential participants were informed that participation was voluntary and that refusal would not lead to any negative consequences. After scanning the QR code, individuals first viewed an electronic informed consent form and could proceed to the questionnaire only after indicating agreement. Because the anonymous QR-code survey was disseminated through open communication channels, the total number of healthcare professionals who received or viewed the invitation could not be accurately determined, and a formal response rate could not be calculated.

### Data collection and quality control

Participants were informed that participation was voluntary and that refusal would not result in any adverse consequences. After scanning the QR code, participants first viewed an electronic informed consent form and could access the questionnaire only after selecting “agree.” Each device or IP address was restricted to a single submission to reduce duplicate responses. The electronic questionnaire platform was configured to require responses for all items, and submissions with missing answers were not permitted; therefore, all analyzed questionnaires were complete, and no missing data were present for the study variables.

Four criteria were applied to identify and exclude invalid responses: (1) failure on an embedded attention-check item (K14: “Please select ‘b. Have heard of it’”); (2) completion time of less than 60 s; (3) implausible demographic values (e.g., age >100); and (4) invariant single-choice response patterns (e.g., selecting the same option for all items).

### Statistical analysis

Statistical analyses were performed using SPSS 26.0 (IBM, Armonk, NY, USA) and AMOS 24.0 (IBM, Armonk, NY, USA). Continuous variables are expressed as means ± standard deviations. Between-group comparisons used independent-samples t-tests or one-way ANOVA when normality was satisfied; the Wilcoxon-Mann–Whitney and Kruskal-Wallis tests were applied as non-parametric alternatives when distributional assumptions were violated. Spearman correlation coefficients quantified associations among KAP dimension scores.

Multivariable logistic regression identified demographic and professional factors independently associated with practice level. Practice scores were dichotomized at ≥70% of maximum (≥24.5 points) following established KAP conventions (21); variables with *p* < 0.05 on univariate analysis were entered into the multivariable model. SEM, using maximum likelihood estimation (MLE), with weighted least squares mean and variance adjusted (WLSMV) estimation applied for robustness verification, was used to examine whether attitude mediated the knowledge-practice relationship. These two analytic approaches served complementary rather than overlapping purposes: logistic regression identified demographic predictors of dichotomized practice, while SEM quantified inter-construct pathways using continuous scores. Ordinal scale items were treated as continuous variables in the SEM, consistent with established methodological guidance (22–24). Model fit was evaluated using RMSEA (<0.08), SRMR (<0.08), CMIN/DF (1-5), IFI, TLI, and CFI (all >0.8). All statistical tests were two-sided, with *p* < 0.05 considered statistically significant. For the main inferential analyses, effect sizes were reported as odds ratios with 95% confidence intervals (logistic regression) and standardized path coefficients with confidence intervals (SEM), providing an estimate of the magnitude and direction of associations. Exploratory between-group comparisons in Table 1 were conducted to describe score distributions across subgroups; given the large number of variables and their descriptive intent, separate effect sizes were not calculated for each comparison.

**Table 1:** Characteristics of the participants and KAP scores.

Variables	*n* (%)	Knowledge (mean ± SD)	*p*	Attitude (mean ± SD)	*p*	Practice (mean ± SD)	*p*
*N* = 401		14.35 ± 5.95		42.63 ± 5.92		24.96 ± 8.93	
Gender			0.163		0.162		0.089
Male	105 (26.18)	14.90 ± 6.09		41.80 ± 6.29		26.20 ± 6.27	
Female	296 (73.82)	14.15 ± 5.90		42.93 ± 5.77		24.52 ± 7.11	
Age			0.132		0.025		0.435
20–30 years	98 (24.44)	14.94 ± 6.02		41.42 ± 6.95		25.63 ± 6.57	
30–40 years	184 (45.89)	14.64 ± 6.03		43.43 ± 5.85		24.57 ± 7.47	
>40 years	119 (29.68)	13.42 ± 5.71		42.39 ± 4.87		25.02 ± 6.35	
Education level			0.225		0.003		0.021
Associate degree or below	59 (14.71)	15.92 ± 5.52		42.03 ± 4.48		25.47 ± 6.53	
Bachelor’s degree	220 (54.86)	14.05 ± 6.06		43.62 ± 4.59		24.02 ± 7.36	
Master’s degree	98 (24.44)	14.21 ± 5.77		42.31 ± 6.77		26.33 ± 6.24	
Doctoral degree or above	24 (5.99)	13.83 ± 6.43		36.33 ± 10.61		26.71 ± 5.39	
Average monthly income (RMB)			0.415		0.085		0.042
<5,000	50 (12.47)	13.40 ± 6.40		42.24 ± 4.89		23.10 ± 6.81	
5,000–10,000	244 (60.85)	14.63 ± 6.01		43.17 ± 5.32		25.32 ± 6.84	
>10,000	107 (26.68)	14.16 ± 5.60		41.59 ± 7.38		25.01 ± 7.12	
Department			0.004		0.672		0.467
Oncology	45 (11.22)	16.62 ± 6.81		41.24 ± 8.37		26.00 ± 6.47	
ICU	30 (7.48)	16.77 ± 5.32		41.60 ± 7.45		24.77 ± 8.02	
Geriatrics	112 (27.93)	14.11 ± 5.36		43.18 ± 5.23		24.56 ± 6.96	
Emergency	22 (5.49)	14.82 ± 6.16		40.05 ± 8.25		26.59 ± 4.96	
Other	192 (47.88)	13.53 ± 5.96		43.09 ± 4.87		24.79 ± 7.05	
Years of professional experience			0.669		0.618		0.168
≤5	64 (15.96)	14.59 ± 5.97		41.97 ± 6.32		25.47 ± 7.16	
5–10	105 (26.18)	14.58 ± 6.09		42.73 ± 6.45		25.75 ± 6.78	
11–15	103 (25.69)	14.48 ± 6.02		42.72 ± 6.15		23.92 ± 7.21	
≥16	129 (32.17)	13.94 ± 5.82		42.81 ± 5.06		24.89 ± 6.69	
Professional title			0.918		0.571		0.655
No/junior title	141 (35.16)	14.54 ± 6.16		42.56 ± 5.43		24.57 ± 7.28	
Intermediate title	190 (47.38)	14.22 ± 5.89		42.73 ± 6.49		24.93 ± 6.96	
Senior title	70 (17.46)	14.33 ± 5.77		42.51 ± 5.30		25.84 ± 6.11	
Hospital level			0.649		0.348		0.185
Tertiary hospital	327 (81.55)	14.36 ± 5.88		42.90 ± 5.65		25.18 ± 7.04	
Secondary/primary/private hospital	74 (18.45)	14.31 ± 6.31		41.46 ± 6.92		24.00 ± 6.40	
Specialized oncology hospital			<0.001		0.909		0.065
Yes	40 (9.98)	18.15 ± 5.95		42.55 ± 6.01		26.65 ± 6.53	
No	361 (90.02)	13.93 ± 5.81		42.64 ± 5.92		24.77 ± 6.96	
Research-oriented hospital			0.964		0.293		0.564
Yes	286 (71.32)	14.40 ± 5.80		42.99 ± 5.45		24.91 ± 6.95	
No	115 (28.68)	14.23 ± 6.35		41.73 ± 6.91		25.10 ± 6.94	
Teaching hospital			0.053		0.174		0.595
Yes	316 (78.80)	14.12 ± 5.90		43.04 ± 5.44		24.89 ± 7.12	
No	85 (21.20)	15.20 ± 6.12		41.09 ± 7.28		25.22 ± 6.22	
Palliative care services available			0.058		0.011		0.641
Yes	213 (53.12)	15.06 ± 6.12		43.42 ± 5.60		25.20 ± 6.90	
No	188 (46.88)	13.55 ± 5.67		41.73 ± 6.16		24.69 ± 6.99	
Involvement in palliative care			0.003		0.011		0.195
Yes	214 (53.37)	15.22 ± 6.38		43.37 ± 5.33		25.49 ± 6.60	
No	187 (46.63)	13.35 ± 5.26		41.78 ± 6.44		24.36 ± 7.27	
Palliative care training/education			<0.001		0.052		0.032
Yes	248 (61.85)	15.53 ± 5.72		42.93 ± 6.15		25.52 ± 6.71	
No	153 (38.15)	12.44 ± 5.84		42.15 ± 5.51		24.06 ± 7.21	
Prior participation in palliative care family meetings			<0.001		0.651		0.013
Yes	95 (23.69)	17.32 ± 6.11		42.26 ± 7.64		26.66 ± 5.79	
No	306 (76.31)	13.43 ± 5.60		42.75 ± 5.28		24.43 ± 7.18	

KAP, knowledge, attitude, and practice.

## Results

### Characteristics of the participants

Of 493 questionnaires collected, 92 were excluded: 47 for incorrect responses to the attention-check item, 38 for invariant single-choice response patterns, 2 for inadequate consent completion, 2 for completion times of less than 60 s, and 3 for implausible or ineligible age values. A total of 401 valid questionnaires were retained for analysis (valid rate: 81.3%).

The sample comprised predominantly female participants (73.8%) aged 30–40 years (45.9%). Most worked in geriatrics (27.9%) or other departments (47.9%), with nearly one-third having ≥16 years of professional experience (32.2%) and approximately half holding an intermediate professional title (47.4%). The majority were employed in tertiary hospitals (81.6%), research-oriented institutions (71.3%), and teaching hospitals (78.8%). Slightly more than half reported that their institutions provided palliative care services (53.1%) and that they had direct or indirect palliative care experience (53.4%). Approximately 61.9% had received palliative care training or education, while only 23.7% had previously participated in a palliative care family meeting (Table 1). Notably, 61.9% of participants reported prior palliative care training, and 53.4% reported direct or indirect involvement in palliative care, yet only 23.7% had previously participated in a palliative care family meeting. This pattern suggests that many respondents were familiar with palliative care in general but had limited direct experience with structured family meetings.

### KAP scores and item-level responses

The mean scores were 14.35 ± 5.95 for knowledge (55.2% of maximum), 42.63 ± 5.92 for attitude (85.3%), and 24.96 ± 8.93 for practice (71.3%). Using the 70% threshold, 193 (48.1%), 374 (93.3%), and 223 (55.6%) participants met the adequacy criterion for knowledge, attitude, and practice, respectively (Table 1).

Regarding knowledge, general awareness of family meeting concepts was moderate: 68.3% had heard of the definition (K1), though only 20.2% were very familiar with it. Familiarity was relatively higher for communication principles (approximately 70% had heard of plain-language use and emotional facilitation; K7-K8) but lower for procedural and logistical aspects, including recommended meeting duration (K9), avoidance of reactive use in emergencies (K10), and distinction from case discussions (K11), with unfamiliarity rates of 13–16% (Table 2).

**Table 2:** Distribution of responses in the knowledge dimension (*N* = 401).

Knowledge items, *n* (%)	Very familiar	Have heard of it	Not familiar
K1. “Family meetings in palliative care” refer to a form of communication in which a multidisciplinary team discusses care goals together with the patient’s family.	81 (20.20)	274 (68.33)	46 (11.47)
K2. Family meetings should be held when there are changes in patient condition, major medical decisions are required, or when requested by the patient or family.	83 (20.70)	278 (69.33)	40 (9.98)
K3. One primary goal of family meetings is to help patients or family members participate in medical decision-making.	91 (22.69)	271 (67.58)	39 (9.73)
K4. Conducting family meetings requires a multidisciplinary team including physicians, nurses, social workers, and psychologists.	103 (25.69)	257 (64.09)	41 (10.22)
K5. Palliative care nurses often serve as facilitators of family meetings, responsible for organizing, guiding, and summarizing meetings.	80 (19.95)	264 (65.84)	57 (14.21)
K6. Before holding a family meeting, the patient’s family structure and values should be assessed.	72 (17.96)	280 (69.83)	49 (12.22)
K7. During family meetings, team members should explain the patient’s condition and discuss care goals using plain language.	84 (20.95)	281 (70.07)	36 (8.98)
K8. Healthcare professionals should actively encourage family members to express their emotions and concerns.	92 (22.94)	272 (67.83)	37 (9.23)
K9. The recommended duration of a family meeting is generally 30–60 min.	73 (18.20)	265 (66.08)	63 (15.71)
K10. Family meetings should be avoided as a reactive measure during acute or emergency situations.	83 (20.70)	265 (66.08)	53 (13.22)
K11. Family meetings cannot replace case discussions or clinical consultations.	110 (27.43)	244 (60.85)	47 (11.72)
K12. Whether patients with decision-making capacity participate should be based on their consent; for those without capacity, family consent is required.	95 (23.69)	257 (64.09)	49 (12.22)
K13. Family meetings differ from routine medical communication in being structured, goal-oriented, and emphasizing consensus-building and multidisciplinary collaboration.	93 (23.19)	266 (66.33)	42 (10.47)

Attitude scores were consistently high. More than 94% agreed or strongly agreed that family meetings are necessary (A1), improve end-of-life care quality (A2), require multidisciplinary participation (A3), reduce family anxiety (A4), and enhance communication (A5). Although 66.3% expressed some concern about emotional confrontation (A6) and 67.1% perceived potential workload increases (A7), 92.0% expressed willingness to receive related training (A8; Table 3).

**Table 3:** Distribution of responses in the attitude dimension (*N* = 401).

Attitude items, *n* (%)	Strongly agree	Agree	Neutral	Disagree	Strongly disagree
A1. I believe that conducting family meetings in palliative care is highly necessary.	199 (49.63)	178 (44.39)	18 (4.49)	3 (0.75)	3 (0.75)
A2. I believe that family meetings help improve the quality of end-of-life care.	211 (52.62)	169 (42.14)	16 (3.99)	4 (1.00)	1 (0.25)
A3. I believe that multidisciplinary team participation in family meetings is necessary.	210 (52.37)	167 (41.65)	12 (2.99)	7 (1.75)	5 (1.25)
A4. I believe that family meetings can reduce anxiety and distress among family members.	209 (52.12)	169 (42.14)	14 (3.49)	4 (1.00)	5 (1.25)
A5. I believe that family meetings promote communication and mutual understanding between healthcare professionals and patients/families.	217 (54.11)	163 (40.65)	12 (2.99)	4 (1.00)	5 (1.25)
A6. I am concerned that family meetings may trigger emotional confrontation or conflict.	124 (30.92)	142 (35.41)	58 (14.46)	61 (15.21)	16 (3.99)
A7. I believe that family meetings may increase the workload of healthcare professionals.	114 (28.43)	155 (38.65)	55 (13.72)	64 (15.96)	13 (3.24)
A8. If training opportunities are available, I am willing to learn skills related to conducting family meetings.	172 (42.89)	197 (49.13)	27 (6.73)	1 (0.25)	4 (1.00)
A9. I believe that family meetings should be incorporated into routine palliative care practice.	170 (42.39)	198 (49.38)	24 (5.99)	5 (1.25)	4 (1.00)
A10. I believe that family meetings help me better understand patients’ values and care goals.	176 (43.89)	198 (49.38)	20 (4.99)	4 (1.00)	3 (0.75)

In terms of practice, 55.6% reported always or often proactively suggesting family meetings when patient conditions changed (P1). Pre-meeting needs assessment (P2) was performed regularly by 69.8%, while multidisciplinary goal-setting (P3) was reported by only 43.6%. Use of plain language (P4: 71.8%) and emotional facilitation (P5: 63.6%) were relatively common, and most participants documented key discussion points (P6: 64.1%) and engaged in post-meeting follow-up (P7: 58.9%). The most frequently reported implementation barriers (P8) were emotional conflicts among family members (90.3%), patients’ limited capacity to participate (78.3%), and difficulties in multidisciplinary coordination (68.6%; Table 4).

**Table 4:** Distribution of responses in the practice dimension (*N* = 401).

Practice items, *n* (%)	Always	Often	Sometimes	Occasionally	Never
P1. I proactively suggest holding a family meeting when the condition of a terminally ill patient changes.	89 (22.19)	134 (33.42)	81 (20.20)	64 (15.96)	33 (8.23)
P2. I assess family members’ understanding and needs before the family meeting.	110 (27.43)	170 (42.39)	56 (13.97)	41 (10.22)	24 (5.99)
P3. I participate in setting meeting goals together with a multidisciplinary team.	63 (15.71)	112 (27.93)	88 (21.95)	76 (18.95)	62 (15.46)
P4. I communicate with family members using plain language rather than medical jargon.	120 (29.93)	168 (41.90)	50 (12.47)	49 (12.22)	14 (3.49)
P5. I actively encourage family members to express their emotions and concerns.	95 (23.69)	160 (39.90)	65 (16.21)	55 (13.72)	26 (6.48)
P6. I document key discussion points and agreed-upon care decisions after the meeting.	102 (25.44)	155 (38.65)	62 (15.46)	52 (12.97)	30 (7.48)
P7. I actively participate in post-meeting follow-up care.	91 (22.69)	145 (36.16)	71 (17.71)	59 (14.71)	35 (8.73)

### Multivariable analysis of factors associated with practice

Multivariable logistic regression identified knowledge score (OR = 1.088, 95%CI: 1.046–1.132, *p* < 0.001), attitude score (OR = 1.063, 95%CI: 1.023–1.104, *p* = 0.002), and monthly income <5,000 RMB versus >10,000 RMB (OR = 0.448, 95%CI: 0.217–0.923, *p* = 0.029) as independent associates of proactive practice toward palliative care family meetings (Table 5).

**Table 5:** Multivariable logistic regression analysis of factors independently associated with proactive practice.

Variable	OR (adjusted)	95% CI	*p*-value
Variables with *p* < 0.05 on univariate analysis were entered into the multivariable model. Only independent associates are presented.			
Knowledge score (continuous)	1.088	1.046–1.132	<0.001
Attitude score (continuous)	1.063	1.023–1.104	0.002
Monthly income: <5,000 vs. >10,000 RMB	0.448	0.217–0.923	0.029

Adjusted OR = odds ratio adjusted for all variables with *p* < 0.05 on univariate analysis.

### Correlation and SEM analyses

Spearman correlation analysis revealed significant positive associations between knowledge and attitude (*r* = 0.239, *p* < 0.001), knowledge and practice (*r* = 0.317, *p* < 0.001), and attitude and practice (*r* = 0.320, *p* < 0.001; Supplementary Table S3).

**Figure 1:**
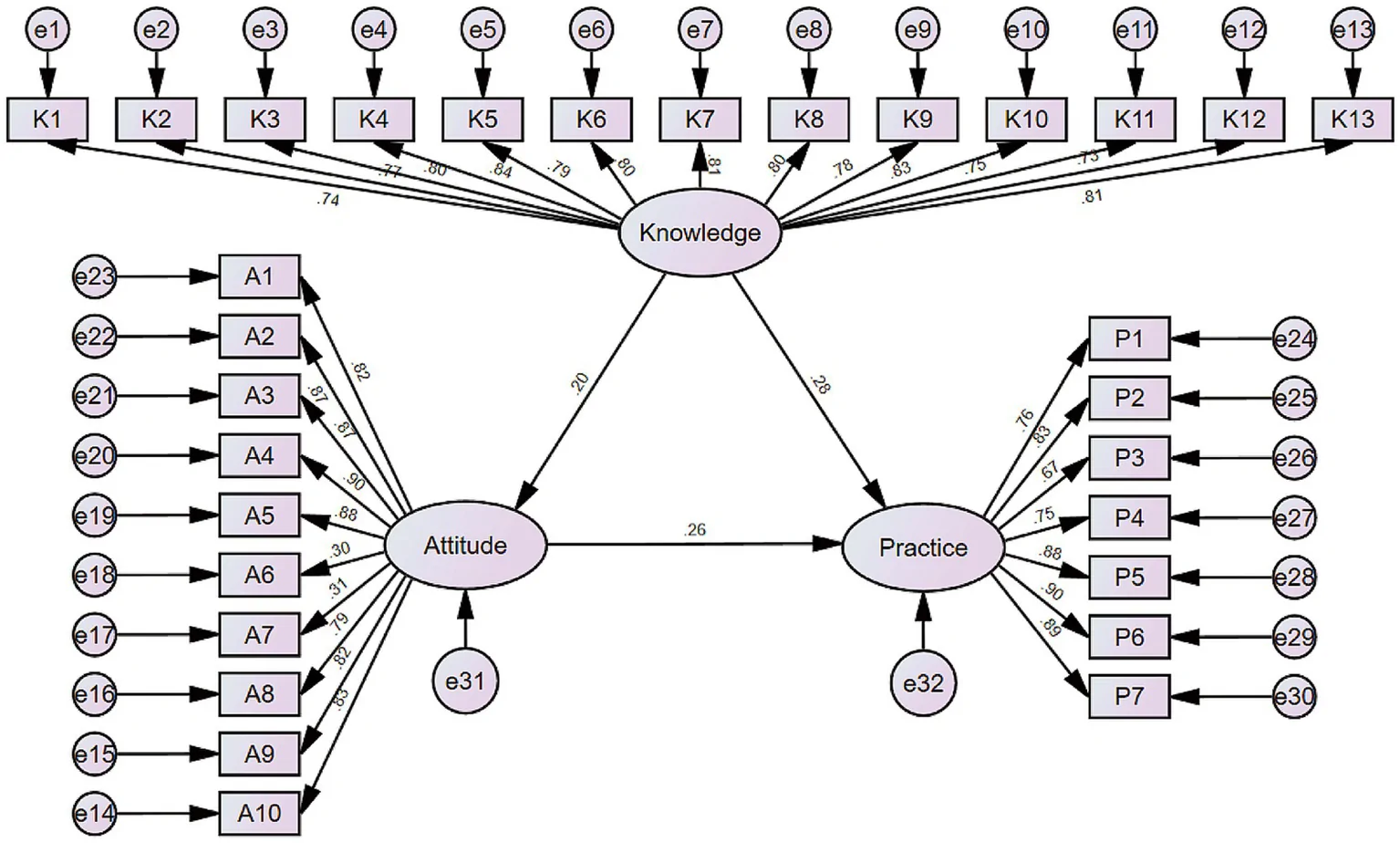
Structural equation model depicting the relationships among knowledge, attitude, and practice. Unidirectional arrows indicate the direction of postulated causal effects. Standardized path coefficients (*β*) and statistical significance are shown. The figure was created using AMOS software.

The SEM model demonstrated acceptable fit under MLE (RMSEA = 0.072, SRMR = 0.050, CMIN/DF = 3.062, IFI = 0.916, TLI = 0.909, CFI = 0.916), with WLSMV estimation yielding comparable results (Supplementary Table S4). As shown in Figure 1, knowledge was significantly associated with attitude (standardized *β* = 0.204, 95%CI: 0.100–0.318, *p* = 0.003). Knowledge exerted a significant total effect on practice (β = 0.329, 95%CI: 0.219–0.417, *p* = 0.009), comprising a direct effect (*β* = 0.277, 95%CI: 0.174–0.378, *p* = 0.010) and an indirect effect mediated through attitude (*β* = 0.052, 95%CI: 0.024–0.107, *p* = 0.003). Attitude also exerted a significant direct effect on practice (*β* = 0.256, 95%CI: 0.143–0.348, *p* = 0.015; Supplementary Tables S5, S6).

## Discussion

This multicenter cross-sectional study examined healthcare professionals’ KAP regarding palliative care family meetings in Fujian Province, China. The findings showed a clear pattern: participants reported positive attitudes and moderately proactive practices, but their perceived knowledge remained limited. In addition, both perceived knowledge and attitude were independently associated with practice, and attitude partially mediated the relationship between perceived knowledge and practice. Together, these findings provide an empirical basis for interventions aimed at strengthening family meeting implementation in palliative care settings.

This pattern suggests that favorable attitudes alone are not sufficient to ensure consistent, high-quality family meeting practice (11, 25, 26). Although many healthcare professionals endorsed the value of family meetings, important gaps remained in procedural and boundary-related knowledge, particularly regarding meeting duration, appropriate timing, and distinctions between family meetings and routine case discussions. These deficits are likely to hinder structured implementation even when clinicians are supportive of the concept. This interpretation is consistent with prior literature showing that healthcare professionals may value family meetings in principle but still lack the practical knowledge needed for protocol-driven delivery (7, 11, 27). Although most participants had received palliative care training and reported involvement in palliative care, only a small proportion had previously participated in family meetings. This suggests that prior training may have focused mainly on general palliative care concepts and symptom management, with relatively less emphasis on structured, skills-based training in conducting family meetings. Such a training profile is consistent with the combination of high attitudinal support and limited procedural knowledge observed in this study and may help explain why proactive practice was moderate rather than consistently high. Clinically, these findings indicate that future educational programs should explicitly incorporate practical, scenario-based training on family meeting preparation, facilitation, and follow-up, rather than assuming that general palliative care training will automatically translate into competence in this specific communication modality.

These findings advance the conceptual understanding of KAP dynamics in palliative settings. The SEM findings indicate that higher knowledge scores are statistically associated with more proactive practice and more positive attitudes within the proposed theoretical framework. These associations are consistent with the hypothesized pathway whereby greater familiarity with family meeting concepts and procedures coexists with more favorable attitudes and more frequent engagement in related practices. However, because the data are cross-sectional, these relationships should be interpreted as associative rather than strictly causal, and the SEM model is best viewed as hypothesis-generating rather than confirmatory. In this context, knowledge can be understood as a key correlate of practice, rather than a proven causal determinant. This pattern is consistent with KAP studies in related palliative care domains, where higher knowledge scores have been associated with more frequent use of palliative consultation services and stronger hospice care preferences (28, 29). Practically, this implies that educational interventions should prioritize building procedural and factual knowledge, not merely promoting favorable attitudes, if the goal is to achieve sustained behavioral change.

An important and somewhat counterintuitive finding is that perceived knowledge scores remained limited even though nearly two-thirds of participants reported prior palliative care training and more than half reported involvement in palliative care. Several factors may help explain this pattern. First, the training that participants received may have been brief, occurred some time ago, or varied in quality and depth, limiting its impact on sustained procedural knowledge. Second, many respondents may have had few opportunities to apply palliative care skills, particularly structured family meetings, in their daily practice, which could lead to attrition of specific knowledge over time. Third, the knowledge dimension in this study primarily assessed familiarity with concrete aspects of family meetings (such as meeting structure, procedures, timing, and role allocation), whereas previous training may have focused more on general palliative care principles, symptom management, or ethical issues. In such cases, general training might not translate directly into detailed, protocol-level knowledge of how to plan and conduct family meetings.

Income-related disparities in practice behavior merit particular attention. Healthcare professionals earning less than 5,000 RMB per month were significantly less likely to engage in proactive family meeting practices (OR = 0.448), an association that likely reflects broader structural inequities in lower-resource institutions, including limited training access, greater workload constraints, reduced multidisciplinary coordination capacity, and fewer organizational supports for end-of-life communication. These findings align with evidence linking under-resourced clinical environments to lower adherence to palliative care best practices (4, 6, 30), suggesting that educational investment alone is insufficient: structural and financial enablers are equally essential for translating knowledge into consistent practice. Additionally, the cultural context of family-centered decision-making in China, including the role of families in mediating prognostic disclosure and making collective end-of-life decisions, requires that international communication frameworks be adapted to accommodate relational norms while simultaneously safeguarding patient autonomy and informed consent. Future interventions should therefore integrate culturally responsive communication models that honor these dual imperatives without compromising ethical standards.

These findings can be understood in relation to established international models and guidelines for family meetings in palliative care (5, 7, 31). Multidisciplinary clinical practice guidelines and systematic reviews from high-income settings highlight several core components of effective family meetings, including structured pre-meeting preparation, clear definition of team roles, goal-oriented agendas, use of plain language, active emotional support for families, systematic documentation of key discussion points, and planned follow-up after the meeting (5, 7, 31). In the present study, many participants reported positive attitudes toward multidisciplinary involvement and communication, and relatively frequent use of plain language, emotional facilitation, documentation, and follow-up, suggesting partial alignment with these recommended practices. However, lower familiarity with logistical and boundary-related elements, such as recommended meeting duration, appropriate timing relative to clinical urgency, and distinctions between family meetings and routine case discussions, indicates that current practice in this middle-income context may not yet fully reflect the structured, protocol-driven approach envisioned in these international models. Together, these comparisons underscore the need to adapt and disseminate evidence-based family meeting frameworks in ways that are feasible and culturally appropriate for Chinese palliative care settings.

The observed KAP patterns also resonate with and extend insights from foundational family meeting and palliative care communication literature. Early models of family conferences in palliative settings described structured, phased approaches that include pre-meeting preparation, agenda-setting, exploration of patient and family values, delivery of information in clear, empathic language, and explicit documentation of decisions and follow-up plans (4, 32). Subsequent communication frameworks have emphasized the importance of defined multidisciplinary roles, attention to emotional dynamics within the family, and deliberate strategies to support surrogate decision-makers under stress (5–7). In the current study, healthcare professionals reported strong attitudinal endorsement of multidisciplinary participation and emotional support, and relatively frequent use of documentation and follow-up, which are consistent with these foundational models. At the same time, lower familiarity with specific procedural parameters, such as meeting duration, timing relative to clinical urgency, and distinctions between family meetings and routine case discussions, suggests that core structural elements identified in early family meeting literature are not yet fully embedded in everyday practice in this setting.

The present study has practical implications for both clinical practice and health policy. At the institutional level, family meetings may be more consistently implemented when they are embedded within formal palliative care pathways, supported by clear protocols, protected time, defined multidisciplinary roles, and relevant quality indicators. At the educational level, training should emphasize not only positive attitudes but also concrete procedural knowledge and communication skills, including preparation, facilitation, documentation, and follow-up. Case-based simulation and interprofessional training may be especially useful for translating conceptual understanding into routine practice. Taken together, these associative pathways suggest that interventions targeting both knowledge and attitudes may be useful for promoting more consistent family meeting practice, although longitudinal or interventional research is required to determine whether changes in these constructs lead to sustained changes in behavior.

The questionnaire used in this study demonstrated excellent internal consistency across the knowledge, attitude, and practice domains, with Cronbach’s alpha values exceeding 0.90 for several subscales. While such values indicate strong internal reliability, they may also suggest some degree of item redundancy, particularly within the knowledge and practice dimensions. This pattern underscores that the present instrument should be viewed as a preliminary, yet rigorously developed, tool for assessing KAP regarding family meetings, and that further psychometric refinement is warranted. Future work could explore item reduction to improve efficiency while retaining content coverage, as well as test–retest reliability and measurement invariance analyses across different professional groups (e.g., physicians, nurses, social workers, psychologists) to ensure stability and comparability of scores.

### Limitations

This study has several limitations. First, both the participating hospitals and individual healthcare professionals were recruited using convenience sampling, and the anonymous QR-code survey was distributed through open channels, which precluded accurate determination of the number of individuals reached and a formal response rate. As a result, the sample may be subject to selection bias and may over-represent healthcare professionals who are more engaged with palliative care activities or more comfortable with electronic surveys. In addition, all four participating hospitals were located in a single province and were selected through an existing regional collaboration network based on survey feasibility. Most respondents were from tertiary, teaching, or research-oriented hospitals, which may have overrepresented healthcare professionals working in relatively resource-rich settings with greater access to palliative care training, multidisciplinary collaboration, and established services. Consequently, professionals from community, township, county-level, rural, non-teaching, private, and long-term care institutions, as well as settings with less developed palliative care services, may have been underrepresented, limiting the generalizability of the findings. In addition, 47.9% of participants came from a heterogeneous “other departments” category, which may have obscured specialty-specific differences in KAP regarding palliative care family meetings. Although the sample included multiple professional groups (physicians, nurses, pharmacists, rehabilitation therapists, psychologists, and social workers), the distribution across these categories was imbalanced, with relatively small numbers in some professions. Consequently, profession-specific subgroup analyses were not conducted, and potential differences in knowledge, attitudes, and practices across professional roles could not be examined in a statistically robust manner. Future research should aim to recruit larger and more balanced samples across disciplines to enable meaningful subgroup comparisons and to inform profession-specific training and implementation strategies for palliative care family meetings. Second, the cross-sectional design of this study does not permit firm causal inference. Although the SEM framework specifies directional paths from knowledge to attitude and practice, these paths represent statistically estimated associations based on the hypothesized model structure, not empirically verified temporal or causal effects. The observed relationships among knowledge, attitude, and practice should therefore be interpreted as exploratory and hypothesis-generating. Third, all measures were self-reported, which may be subject to social desirability and recall bias; additionally, the knowledge scale primarily captured perceived familiarity rather than objective competence, and the 70% threshold for classifying “good” KAP, while widely used, remains operationally arbitrary (13, 21). Different cut-offs could alter the proportions classified as adequate and should be taken into account when interpreting the categorical findings. Fourth, although the questionnaire demonstrated strong internal consistency overall, with Cronbach’s alpha values above 0.90 for some domains, such high coefficients may indicate redundancy among items and a relatively narrow construct representation. Other aspects of instrument validation, including test–retest reliability, responsiveness to change, and measurement invariance across professional subgroups, were not assessed in this study. As a result, the KAP scores should be interpreted with some caution, and the present findings should be considered as an initial psychometric evaluation rather than definitive evidence of instrument robustness. Future validation studies are needed to refine the item set, confirm stability over time, and verify that the scale functions equivalently across different categories of healthcare professionals. Fifth, although the final analysis set (*n* = 401) exceeded the minimum required sample size (n = 385), the exclusion of 92 of 493 collected questionnaires (exclusion rate: 18.7%, valid response rate: 81.3%) may have introduced some selection bias toward more attentive or engaged respondents, potentially inflating observed knowledge and practice scores. Sixth, despite multivariable adjustment and SEM, residual confounding remains possible; the observed associations among knowledge, attitude, and practice should therefore be interpreted as exploratory and hypothesis-generating rather than confirmatory. Finally, it is also important to recognize that the knowledge scale in this study measured self-reported familiarity rather than objective knowledge. Participants were asked whether they had “heard of” or were “very familiar” with specific concepts and procedural elements of family meetings, but their understanding was not formally tested through case vignettes, scenario-based questions, or performance assessments. As a result, lower knowledge scores may reflect cautious self-appraisal or lack of confidence in detailed protocol-level knowledge, even when participants are able to implement some core elements of family meetings in practice. This measurement characteristic may partly explain the apparent discrepancy between limited knowledge scores and relatively positive practice scores and should be taken into account when interpreting the findings.

### Future perspectives

Future studies should consider probability-based or stratified sampling across a wider range of institutions, including community and rural settings, and a more granular classification of clinical departments to better capture specialty-level variation and enhance external validity. Although this study captured perspectives from several professional groups involved in palliative care family meetings, the imbalanced subgroup sizes limited the ability to formally compare KAP patterns across disciplines; future work should explore such differences to guide tailored educational interventions.

## Conclusion

This multicenter cross-sectional study found that healthcare professionals in Fujian Province demonstrated positive attitudes and moderately proactive practices toward palliative care family meetings, but had limited knowledge. Both perceived knowledge and attitude were independently associated with practice level, with attitude partially mediating the knowledge-practice relationship. These findings highlight the need for systematic, knowledge-focused training programs, complemented by institutional support including structured protocols, protected time, and multidisciplinary coordination mechanisms, to translate positive attitudinal endorsement into consistent, high-quality family meeting practice. These considerations suggest that future educational initiatives should include dedicated, skills-based modules on family meetings and ensure that participants have opportunities to practice and reinforce these competencies in real clinical settings.

## Data Availability

The original contributions presented in the study are included in the article/Supplementary material, further inquiries can be directed to the corresponding authors.

## Supplementary material

SUPPLEMENTARY FIGURE S1 Confirmatory factor analysis.
