# Supplementary material for: Knowledge, attitudes, and practices of healthcare professionals regarding palliative care family meetings: a multicenter cross-sectional study
Source: Front Public Health. 2026 Aug 21;14:1902540. doi: 10.3389/fpubh.2026.1902540 (PMC13541703; doi:10.3389/fpubh.2026.1902540)
Supplement: Supplementary file 1 [file Supplementary_file_1.DOCX]

|  | Questionnaire ID: | | |
| --- | --- | --- | --- |
|  | Dear Participant,  We are a research team from Fujian Provincial Hospital Affiliated to Fuzhou University. We sincerely invite you to participate in our research study. This study aims to explore healthcare professionals’ knowledge, attitudes, and practices regarding family meetings in palliative care, in order to provide evidence for developing scientifically sound intervention strategies. These strategies may help more people and improve their health outcomes in the future. Your participation in this study is entirely voluntary. This study has been reviewed and approved by the Ethics Committee. If you agree to participate, please read the following information carefully:  1. Please complete the questionnaire according to your actual situation. There are no right or wrong answers. If you have any questions during the process, you may contact the researchers at any time. Please submit the questionnaire after completion.  2. This study is a simple questionnaire survey and will not cause any physical or psychological harm. However, it involves some personal information (such as gender and age). All collected information will be kept strictly confidential and will not be disclosed. Please feel free to respond honestly.  3. As a participant, you have the right to access information related to this study and its progress at any time. If you decide to withdraw from the study, please inform us, and your data will not be included in the final analysis.  Finally, we sincerely thank you for taking the time to support our scientific research!  I have read and understood the above information and agree that the collected data may be used for scientific research purposes.  Informed Consent Signature:  Date of Participation: ______ Year ______ Month ______ Day | | |
| **Part 1 Basic Information** | | |  |
|  | | |  |
| 1. **Your gender:** | | a. Male b. Female |  |
| 2. **Your age: ______ years (Please set integer validation in the online questionnaire system)** | | |  |
| 3. **Your highest level of education:** | | a. Associate degree or below b. Bachelor’s degree c. Master’s degree d. Doctoral degree or above |  |
| 4. **Your average monthly income (RMB):** | | a.<2000  b.2000-5000  c.5000-10000  d.10000-20000  e.>20000 |  |
| 5. **Your department:** | | a. Oncology b. Intensive Care Unit (ICU) c. Geriatrics d. Emergency Department e. Others |  |
| 6. **Your profession:** | | a. Physician b. Nurse c. Pharmacist d. Nutritionist/Dietitian e. Rehabilitation therapist f. Psychological counselor g. Social worker h. Others |  |
| 7. **Your professional title:** | | a. No professional title b. Junior title c. Intermediate title d. Senior title (including associate senior and senior titles) |  |
| 8. **Years of professional experience:** | | a. ≤ 5 years b. 5–10 years c. 11–15 years d. ≥ 16 years |  |
| 9. **The level of your hospital:** | | a. Tertiary hospital b. Secondary hospital c. Primary hospital d. Private hospital |  |
| 10. **Is your medical institution a specialized oncology hospital?** | | a. Yes b. No |  |
| 11. **Is your hospital a teaching hospital?** | | a. Yes b. No |  |
| 12. **Is your hospital a research-oriented hospital?** | | a. Yes b. No |  |
| 13. **Does your medical institution provide palliative care services?** | | a. Yes b. No |  |
| 14. **In your professional work, have you been directly or indirectly involved in palliative care?** | | a. Yes b. No |  |
| 15. **In the past year, have you participated in any training or educational lectures related to palliative care?** | | a. Yes b. No |  |
| 16. **In the past year, have you participated in family meetings in palliative care?** | | a. Yes b. No |  |

| **Part 2 Knowledge of Family Meetings in Palliative Care** | | | |  |
| --- | --- | --- | --- | --- |
| 1. **“Family meetings in palliative care” refer to a form of communication in which a multidisciplinary team discusses care goals together with the patient’s family.** | a. very familiar | b. have heard of it | c. not familiar | |
| 2. **Family meetings should be held when there are changes in the patient’s condition, major medical decisions need to be made, or when requested by the patient or family members.** | a. very familiar | b. have heard of it | c. not familiar | |
| 3. **One of the primary goals of family meetings is to help patients or family members participate in medical decision-making.** | a. very familiar | b. have heard of it | c. not familiar | |
| 4. **Conducting family meetings requires a multidisciplinary team, including physicians, nurses, social workers, and psychological counselors.** | a. very familiar | b. have heard of it | c. not familiar | |
| 5. **Palliative care nurses often serve as facilitators of family meetings and are responsible for organizing, guiding, and summarizing the meetings.** | a. very familiar | b. have heard of it | c. not familiar | |
| 6. **Before holding a family meeting, the patient’s family structure and values should be assessed.** | a. very familiar | b. have heard of it | c. not familiar | |
| 7. **During family meetings, team members should explain the patient’s condition and discuss care goals and plans with patients and family members using plain and understandable language.** | a. very familiar | b. have heard of it | c. not familiar | |
| 8. **Healthcare professionals should actively encourage family members to express their emotions and concerns to enhance the effectiveness of communication during family meetings.** | a. very familiar | b. have heard of it | c. not familiar | |
| 9. **The recommended duration of a family meeting is generally 30–60 minutes and should not be excessively long.** | a. very familiar | b. have heard of it | c. not familiar | |
| 10. **Family meetings should be avoided as a reactive measure during acute or emergency situations.** | a. very familiar | b. have heard of it | c. not familiar | |
| 11. **Family meetings cannot replace case discussions or clinical consultations.** | a. very familiar | b. have heard of it | c. not familiar | |
| 12. **Whether patients with decision-making capacity participate in family meetings should be based on their consent; for patients without such capacity, consent should be obtained from family members.** | a. very familiar | b. have heard of it | c. not familiar | |
| 13. **Family meetings differ from routine medical communication in that they are structured, goal-oriented, and emphasize consensus-building and multidisciplinary collaboration.** | a. very familiar | b. have heard of it | c. not familiar | |
| 14. **Please select “b. Have heard of it” for this item.** | a. very familiar | b. have heard of it | c. not familiar | |

| **Part 3 Attitudes Toward Family Meetings in Palliative Care** | | | | | |
| --- | --- | --- | --- | --- | --- |
| 1. **I believe that conducting family meetings in palliative care is highly necessary.** | a. strongly agree | b. agree | c. neutral | d. disagree | e. strongly disagree |
| 2. **I believe that family meetings help improve the quality of care for patients at the end of life.** | a. strongly agree | b. agree | c. neutral | d. disagree | e. strongly disagree |
| 3. **I believe that the participation of a multidisciplinary team in family meetings is necessary.** | a. strongly agree | b. agree | c. neutral | d. disagree | e. strongly disagree |
| 4. **I believe that family meetings can reduce anxiety and distress among family members.** | a. strongly agree | b. agree | c. neutral | d. disagree | e. strongly disagree |
| 5. **I believe that family meetings can promote communication and mutual understanding between healthcare professionals and patients/families.** | a. strongly agree | b. agree | c. neutral | d. disagree | e. strongly disagree |
| 6. **I am concerned that family meetings may trigger emotional confrontation or conflict among patients or family members.** | a. strongly agree | b. agree | c. neutral | d. disagree | e. strongly disagree |
| 7. **I believe that family meetings may increase the workload of healthcare professionals.** | a. strongly agree | b. agree | c. neutral | d. disagree | e. strongly disagree |
| 8. **If training opportunities are available, I am willing to learn skills related to conducting family meetings.** | a. strongly agree | b. agree | c. neutral | d. disagree | e. strongly disagree |
| 9. **I believe that family meetings should be incorporated into routine palliative care practice.** | a. strongly agree | b. agree | c. neutral | d. disagree | e. strongly disagree |
| 10. **I believe that family meetings help me better understand patients’ values and care goals.** | a. strongly agree | b. agree | c. neutral | d. disagree | e. strongly disagree |

| **Part 4 Practices Related to Family Meetings in Palliative Care** | | | | | |
| --- | --- | --- | --- | --- | --- |
| 1. **I proactively suggest holding a family meeting when the condition of a terminally ill patient changes.** | a. always | b. often | c. sometimes | d. occasionally | e. never |
| 2. **I assess family members’ understanding and needs before the family meeting.** | a. always | b. often | c. sometimes | d. occasionally | e. never |
| 3. **I have participated in setting meeting goals together with a multidisciplinary team.** | a. always | b. often | c. sometimes | d. occasionally | e. never |
| 4. **I communicate with family members using plain language rather than medical jargon.** | a. always | b. often | c. sometimes | d. occasionally | e. never |
| 5. **I actively encourage family members to express their emotions and concerns during the meeting.** | a. always | b. often | c. sometimes | d. occasionally | e. never |
| 6. **I document key discussion points and consensus outcomes from family meetings.** | a. always | b. often | c. sometimes | d. occasionally | e. never |
| 7. **I participate in follow-up care for patients and family members after the meeting.** | a. always | b. often | c. sometimes | d. occasionally | e. never |
| 8. **Based on your participation in or understanding of family meetings in palliative care, which of the following are the most common difficulties or barriers during implementation? (Multiple answers allowed)**   a. Emotional conflicts or disagreements among family members **(difficulty reaching consensus)** b. Patients unable to participate effectively **(e.g., impaired consciousness or critical condition)** c. Difficulties in multidisciplinary team coordination **(time conflicts or insufficient participation)** d. Lack of standardized procedures **(unclear meeting goals or disorganized processes)** e. Inadequate communication of information **(insufficient understanding of the condition or care plans by family members)** f. Lack of relevant training among healthcare professionals **(insufficient communication or facilitation skills)** g. Inadequate follow-up **(decisions made during meetings not implemented or not tracked)** h. **Physicians’ limited recognition of the value of family meetings, resulting in low willingness to participate** | | | | | |
