# Supplementary material for: Knowledge, attitudes, and practices of healthcare professionals regarding palliative care family meetings: a multicenter cross-sectional study
Source: Front Public Health. 2026 Aug 21;14:1902540. doi: 10.3389/fpubh.2026.1902540 (PMC13541703; doi:10.3389/fpubh.2026.1902540)
Supplement: Supplementary file 2 [file Supplementary_file_2.DOCX]

**Supplementary Table S1.** Fit indexes of the CFA

| Indicators | Reference | Actual |
| --- | --- | --- |
| CMIN/DF | 1-3: Excellent, 3-5: Good | 3.062 |
| RMSEA | <0.08: Good | 0.072 |
| IFI | >0.8: Good | 0.916 |
| TLI | >0.8: Good | 0.909 |
| CFI | >0.8: Good | 0.916 |

CMIN/DF: chi-square value divided by its degrees of freedom; RMSEA: root mean square error of approximation; IFI: incremental fit index; TLI: Tucker-Lewis index; CFI: comparative fit index.

**Supplementary Table S2.** Factor loadings of the CFA.

|  |  |  | Estimate | S.E. | C.R. | P |
| --- | --- | --- | --- | --- | --- | --- |
| K13 | <--- | Knowledge | 1 |  |  |  |
| K12 | <--- | Knowledge | 0.94 | 0.057 | 16.546 | <0.001 |
| K11 | <--- | Knowledge | 0.991 | 0.058 | 17.085 | <0.001 |
| K10 | <--- | Knowledge | 1.052 | 0.053 | 19.858 | <0.001 |
| K9 | <--- | Knowledge | 0.995 | 0.055 | 18.148 | <0.001 |
| K8 | <--- | Knowledge | 0.964 | 0.051 | 18.773 | <0.001 |
| K7 | <--- | Knowledge | 0.941 | 0.05 | 18.951 | <0.001 |
| K6 | <--- | Knowledge | 0.957 | 0.051 | 18.775 | <0.001 |
| K5 | <--- | Knowledge | 1.002 | 0.055 | 18.328 | <0.001 |
| K4 | <--- | Knowledge | 1.057 | 0.053 | 19.947 | <0.001 |
| K3 | <--- | Knowledge | 0.972 | 0.052 | 18.81 | <0.001 |
| K2 | <--- | Knowledge | 0.913 | 0.052 | 17.717 | <0.001 |
| K1 | <--- | Knowledge | 0.893 | 0.054 | 16.668 | <0.001 |
| A1 | <--- | Attitude | 1 |  |  |  |
| A2 | <--- | Attitude | 1.002 | 0.046 | 21.558 | <0.001 |
| A3 | <--- | Attitude | 1.157 | 0.054 | 21.536 | <0.001 |
| A4 | <--- | Attitude | 1.167 | 0.051 | 23.012 | <0.001 |
| A5 | <--- | Attitude | 1.13 | 0.051 | 22.036 | <0.001 |
| A6 | <--- | Attitude | 0.617 | 0.104 | 5.948 | <0.001 |
| A7 | <--- | Attitude | 0.62 | 0.101 | 6.157 | <0.001 |
| A8 | <--- | Attitude | 0.984 | 0.053 | 18.608 | <0.001 |
| A9 | <--- | Attitude | 1.06 | 0.054 | 19.644 | <0.001 |
| A10 | <--- | Attitude | 1.024 | 0.051 | 20.149 | <0.001 |
| P1 | <--- | Practice | 1 |  |  |  |
| P2 | <--- | Practice | 1.016 | 0.057 | 17.747 | <0.001 |
| P3 | <--- | Practice | 0.945 | 0.068 | 13.926 | <0.001 |
| P4 | <--- | Practice | 0.881 | 0.056 | 15.746 | <0.001 |
| P5 | <--- | Practice | 1.042 | 0.055 | 19.006 | <0.001 |
| P6 | <--- | Practice | 1.14 | 0.058 | 19.675 | <0.001 |
| P7 | <--- | Practice | 1.134 | 0.058 | 19.395 | <0.001 |

**Supplementary Table S3.** Correlation analysis

|  | Knowledge | Attitude | Practice |
| --- | --- | --- | --- |
| Knowledge | 1 |  |  |
| Attitude | 0.239 (P<0.001) | 1 |  |
| Practice | 0.317 (P<0.001) | 0.320 (P<0.001) | 1 |

**Supplementary Table S4.** SEM model fit index results

| **Model 1** | **Ref.** | **MLE results** | **WLSMV results** |
| --- | --- | --- | --- |
| RMSEA | <0.08 is good | 0.072 | 0.076 |
| SRMR | <0.08 is good | 0.050 | 0.060 |
| CMIN/DF | 1-3 is excellent, 3-5 is good | 3.062 | - |
| IFI | >0.8 is good | 0.916 | - |
| TLI | >0.8 is good | 0.909 | 0.972 |
| CFI | >0.8 is good | 0.916 | 0.974 |

MLE: maximum likelihood estimation; WLSMV: weighted least squares mean and variance adjusted; RMSEA: root mean square error of approximation; SRMR: standardized root mean square residual; CMIN/DF: chi-square value divided by its degrees of freedom; IFI: incremental fit index; TLI: Tucker-Lewis index; CFI: comparative fit index.

**Supplementary Table S5.** SEM direct and indirect effects.

| **Model paths** | **Standardized total effects** | | **Standardized direct effects** | | **Standardized indirect effects** | |
| --- | --- | --- | --- | --- | --- | --- |
|  | **β (95%CI)** | **P** | **β (95%CI)** | **P** | **β (95%CI)** | **P** |
| Knowledge → Attitude | 0.204 (0.100-0.318) | 0.003 | 0.204 (0.100-0.318) | 0.003 |  |  |
| Knowledge → Practice | 0.329 (0.219-0.417) | 0.009 | 0.277 (0.174-0.378) | 0.010 | 0.052 (0.024-0.107) | 0.003 |
| Attitude → Practice | 0.256 (0.143-0.348) | 0.015 | 0.256 (0.143-0.348) | 0.015 |  |  |

CI: confidence interval.

**Supplementary Table S6.** Path coefficients

|  |  |  | **Estimate** | **S.E.** | **C.R.** | **P** |
| --- | --- | --- | --- | --- | --- | --- |
| Attitude | <--- | Knowledge | 0.285 | 0.074 | 3.879 | <0.001 |
| Practice | <--- | Knowledge | 0.629 | 0.119 | 5.286 | <0.001 |
| Practice | <--- | Attitude | 0.415 | 0.083 | 4.985 | <0.001 |
| K1 | <--- | Knowledge | 1 |  |  |  |
| K2 | <--- | Knowledge | 1.022 | 0.065 | 15.78 | <0.001 |
| K3 | <--- | Knowledge | 1.088 | 0.066 | 16.538 | <0.001 |
| K4 | <--- | Knowledge | 1.184 | 0.068 | 17.295 | <0.001 |
| K5 | <--- | Knowledge | 1.121 | 0.069 | 16.208 | <0.001 |
| K6 | <--- | Knowledge | 1.071 | 0.065 | 16.515 | <0.001 |
| K7 | <--- | Knowledge | 1.053 | 0.063 | 16.634 | <0.001 |
| K8 | <--- | Knowledge | 1.079 | 0.065 | 16.513 | <0.001 |
| K9 | <--- | Knowledge | 1.114 | 0.069 | 16.083 | <0.001 |
| K10 | <--- | Knowledge | 1.177 | 0.068 | 17.237 | <0.001 |
| K11 | <--- | Knowledge | 1.11 | 0.072 | 15.329 | <0.001 |
| K12 | <--- | Knowledge | 1.052 | 0.07 | 14.936 | <0.001 |
| K13 | <--- | Knowledge | 1.119 | 0.067 | 16.668 | <0.001 |
| A10 | <--- | Attitude | 1 |  |  |  |
| A9 | <--- | Attitude | 1.036 | 0.052 | 20.062 | <0.001 |
| A8 | <--- | Attitude | 0.961 | 0.051 | 18.962 | <0.001 |
| A7 | <--- | Attitude | 0.606 | 0.098 | 6.17 | <0.001 |
| A6 | <--- | Attitude | 0.603 | 0.101 | 5.959 | <0.001 |
| A5 | <--- | Attitude | 1.103 | 0.049 | 22.631 | <0.001 |
| A4 | <--- | Attitude | 1.141 | 0.048 | 23.691 | <0.001 |
| A3 | <--- | Attitude | 1.13 | 0.051 | 22.09 | <0.001 |
| A2 | <--- | Attitude | 0.978 | 0.044 | 22.114 | <0.001 |
| A1 | <--- | Attitude | 0.977 | 0.048 | 20.149 | <0.001 |
| P1 | <--- | Practice | 1 |  |  |  |
| P2 | <--- | Practice | 1.016 | 0.057 | 17.747 | <0.001 |
| P3 | <--- | Practice | 0.945 | 0.068 | 13.926 | <0.001 |
| P4 | <--- | Practice | 0.881 | 0.056 | 15.746 | <0.001 |
| P5 | <--- | Practice | 1.042 | 0.055 | 19.006 | <0.001 |
| P6 | <--- | Practice | 1.14 | 0.058 | 19.675 | <0.001 |
| P7 | <--- | Practice | 1.134 | 0.058 | 19.395 | <0.001 |
